# Supplementary material for: The Effect of Statins on Ocular Disorders: A Systematic Review of Randomized Controlled Trials
Source: Pharmaceuticals (Basel). 2023 May 7;16(5):711. doi: 10.3390/ph16050711 (PMC10222124; doi:10.3390/ph16050711)
Supplement: Supplementary file 1 [file pharmaceuticals-16-00711-s001.zip › pharmaceuticals-2360719-supplementary.pdf]

The Effect of Statins on Ocular Disorders: A Systematic Review of Randomized Controlled Trials

Supplementary material

**Supplementary Table S1. PICOS criteria for inclusion and exclusion of studies**		
**Parameter**	**Inclusion**	**Exclusion**
Population	Adult population (>18) receiving statins	Patients not receiving statins, age <18
Intervention	Administration of statins, alone or in conjunction with background treatment	Other interventions
Comparator	Placebo, diet or control group	Statins, Other than placebo/control group
Outcomes	Effect on ocular disorders (Cataract, AMD, glaucoma, diabetic retinopathy, dry eye, uveitis, macular edema)	Other outcomes, or lack of data regarding specific ocular disorders
Study design	Randomized controlled trials, published in English	All other study types,<br>Published in any other language than English

**Supplementary Table S2. Excluded studies with reasons**		
**Study**	**Year**	**Reason of exclusion**
Murakami [1]	2020	Different comparator
Maguire [2]	2009	Not an RCT
Al-Holou [3]	2015	Not an RCT
Chew [4]	2014	Different comparator
Martini*	1991	Language other than English
Gaede [5]	2008	Different comparator
Tobert [6]	1990	Not an RCT

*This study was retrieved after the screening of another systematic review [7]

References

1. Murakami, T.; Kato, S.; Shigeeda, T.; Itoh, H.; Komuro, I.; Takeuchi, M.; Yoshimura, N.; ophthalmology substudy of, E.I. Intensive treat-to-target statin therapy and severity of diabetic retinopathy complicated by hypercholesterolaemia. *Eye (Lond)* **2021**, *35*, 2221-2228, doi:10.1038/s41433-020-01202-5.
2. Maguire, M.G.; Ying, G.S.; McCannel, C.A.; Liu, C.; Dai, Y.; Complications of Age-related Macular Degeneration Prevention Trial Research, G. Statin use and the incidence of advanced age-related macular degeneration in the Complications of Age-related Macular Degeneration Prevention Trial. *Ophthalmology* **2009**, *116*, 2381-2385, doi:10.1016/j.ophtha.2009.06.055.
3. Al-Holou, S.N.; Tucker, W.R.; Agron, E.; Clemons, T.E.; Cukras, C.; Ferris, F.L., 3rd; Chew, E.Y.; Age-Related Eye Disease Study 2 Research, G. The Association of Statin Use with Age-Related Macular Degeneration Progression: The Age-Related Eye Disease Study 2 Report Number 9. *Ophthalmology* **2015**, *122*, 2490-2496, doi:10.1016/j.ophtha.2015.08.028.
4. Chew, E.Y.; Davis, M.D.; Danis, R.P.; Lovato, J.F.; Perdue, L.H.; Greven, C.; Genuth, S.; Goff, D.C.; Leiter, L.A.; Ismail-Beigi, F.; et al. The effects of medical management on the progression of diabetic retinopathy in persons with type 2 diabetes: the Action to Control Cardiovascular Risk in Diabetes (ACCORD) Eye Study. *Ophthalmology* **2014**, *121*, 2443-2451, doi:10.1016/j.ophtha.2014.07.019.
5. Gaede, P.; Lund-Andersen, H.; Parving, H.H.; Pedersen, O. Effect of a multifactorial intervention on mortality in type 2 diabetes. *N Engl J Med* **2008**, *358*, 580-591, doi:10.1056/NEJMoa0706245.
6. Tobert, J.A.; Shear, C.L.; Chremos, A.N.; Mantell, G.E. Clinical experience with lovastatin. *Am J Cardiol* **1990**, *65*, 23F-26F, doi:10.1016/0002-9149(90)91251-z.
7. Gehlbach, P.; Li, T.; Hatef, E. Statins for age-related macular degeneration. *Cochrane Database Syst Rev* **2016**, *2016*, Cd006927, doi:10.1002/14651858.CD006927.pub5.
